# A Retrospective 8-Year Single Institutional Study in Germany Regarding Diagnosis, Treatment, and Outcome of Malignant Parotid Tumors

**DOI:** 10.1155/ijso/7598063

**Published:** 2024-12-09

**Authors:** S. Andrianopoulou, L. S. Fiedler, B. M. Lippert, O. C. Bulut

**Affiliations:** ^1^Department for Otorhinolaryngology, Head and Neck, Plastic Surgery, SLK Clinics, Heilbronn, Baden-Württemberg, Germany; ^2^Medical University Heidelberg, Heidelberg, Baden-Württemberg, Germany

**Keywords:** diagnosis, outcome, parotid cancer, parotidectomy, radiotherapy, survival rate, treatment, tumor

## Abstract

This study sought to comprehensively evaluate the diagnosis, therapeutic interventions, and outcomes of individuals afflicted with malignant parotid tumors at a tertiary care otolaryngology department in Heilbronn, Germany, spanning the years 2010–2018. The primary objective was to juxtapose this dataset with findings from analogous single and multicenter investigations. We conducted a meticulous analysis of electronic medical records pertaining to 45 patients subjected to primary parotid cancer treatment. The male-to-female ratio was 3:2, with an average age of 61 years. Predominant histological types included mucoepidermoid and squamous cell carcinomas, with ultrasound emerging as the predominant diagnostic modality (97.8% sensitivity). Intraoperative frozen sections exhibited a high level of sensitivity. Notably, lymph node metastasis was prevalent in T3 tumors, frequently located intraparotid and at Neck level II. Solely one patient exhibited distant metastases (pulmonary). All patients underwent parotidectomy, and 29% necessitated a secondary procedure due to positive resection margins. Postoperative complications encompassed facial nerve palsy, seromas, and salivary fistulas. Adjuvant radiotherapy (38%) was recommended for high-grade tumors, T3/T4 stage, N+, perineural invasion (PNI), and positive or uncertain surgical margins. Neck dissection was executed in 67% of instances, with 20% revealing occult lymph node metastases. Recurrence manifested in 22% of patients, primarily as locoregional recurrence (80%) and distant metastases (20%). The 3-year recurrence-free survival (RFS), cancer-specific survival (CSS), and overall survival (OS) rates stood at 72.1%, 91.9%, and 87.5%, respectively. Noteworthy factors influencing RFS included preoperative facial palsy, T stage, resection margins, and PNI. In summary, the management of parotid cancer involving surgical interventions, neck dissection, and radiotherapy in high-risk patients yielded commendable outcomes with minimal complications, showcasing survival rates exceeding 70%. Timely diagnosis at an early stage is imperative for achieving tumor-free margins and enhancing survival rates. More assertive therapeutic strategies are advocated for cases presenting with preoperative facial nerve palsy and PNI.

## 1. Introduction

Malignant parotid tumors, constituting approximately 0.5% of all cancers and 1%–5% of head and neck malignancies, pose a rare clinical challenge [1, 2]. Clinical manifestations often mimic those of benign parotid tumors, rendering the differentiation between benign and malignant tumors challenging based on clinical presentation alone. Accurate diagnosis heavily relies on histological examination. Despite the scarcity of German-specific guidelines for managing these tumors, most German clinics, including ours in Heilbronn, adhered to the guidelines established in the UK [3] and the US [4]. A new German guideline was published in May 2024 [5]. Surgical resection remains the cornerstone of treatment. Numerous German and international studies have explored the epidemiology, diagnosis, treatment, outcomes, and prognostic factors of parotid cancer [2, 6–14]. This study aims to scrutinize and assess the diagnostic patterns, treatment modalities, and outcomes of patients treated at our institution in Heilbronn, Germany (population, including suburbs, estimated at 500,000 citizens), and to compare these findings with results from analogous studies.

## 2. Materials and Methods

**Figure 1:**
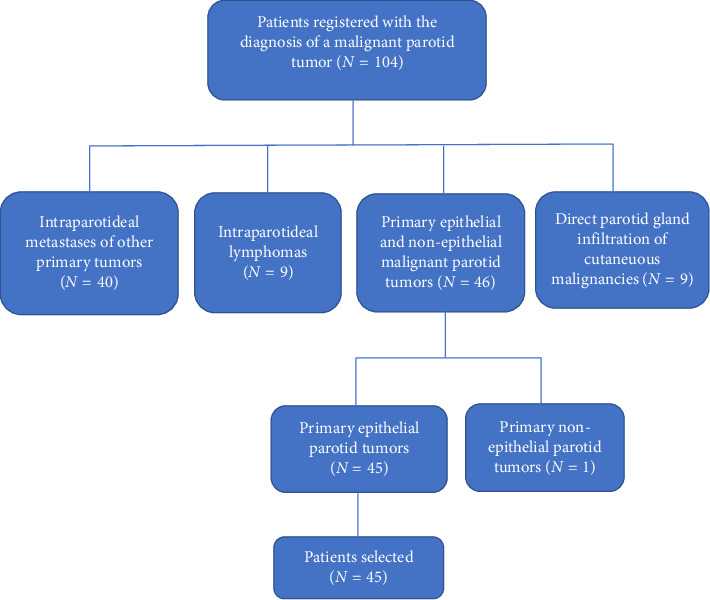
Selection of the patients.

This retrospective study included patients diagnosed with malignant epithelial parotid tumors, admitted to and treated at the department of otolaryngology, head and neck surgery in Heilbronn, Germany, between 2010 and 2018. The selection of the patients was based on the histological entities described in the WHO histological classification of salivary gland tumors of 2017. Excluded were patients with intraparotideal metastases of other primary tumors, parotid lymphomas, nonepithelial parotid tumors, and those with direct gland infiltration from cutaneous malignancies. We decided to focus on the epithelial parotid malignancies because of the rarity of the nonepithelial tumors in our patient collective (solely one patient with a fibrosarcoma) and in the published literature. The patients' selection method is presented in Figure 1. Data were retrospectively collected from electronic medical records, utilizing the international classification of diseases (ICD-10) code C07 (malignant neoplasm of the parotid gland). The median observation period was 43 months, ranging from 1 to 108 months. Statistical analysis involved gender, age at diagnosis, clinical signs and symptoms, diagnostic methods, preoperative diagnosis, type of surgery, histopathological results, TNM staging, grading, resection margins, perineural invasion (PNI), lymphovascular invasion (LVI), molecular biomarkers, location of metastatic lymph nodes, postoperative radiation/chemoradiotherapy, postoperative complications, and recurrence. Survival rates were computed using the Kaplan–Meier method, and survival differences were assessed with the log-rank test. Prognostic factors for lymph node metastases and recurrence were examined using the X2 test. A *p* value < 0.05 was considered statistically significant. Data analysis employed MedCalc Version 19.1 and Microsoft Excel Version 2019 software. The study received approval from the institutional review board in Heidelberg (ethics commission/S-800/2019).

## 3. Results

Among the cohort of 104 patients who underwent surgical intervention for malignant parotid tumors between 2010 and 2018, our study focused on a subset of 45 individuals diagnosed with primary epithelial malignant parotid tumors. Notably, mucoepidermoid and squamous cell carcinomas were the predominant tumor types, collectively accounting for 22.2% of cases. The majority of tumors exhibited a low-grade classification (62.2%), with patients spanning an age range from 11 to 91 years and a mean age of 61 years. The youngest patient in the study was diagnosed with mammary analog secretory carcinoma (MASC). The male-to-female ratio was 3:2, and a palpable lump was discernible in 43 patients.

Clinical presentations varied, revealing 13 cases with rapid growth, 11 with pain, and 5 with facial nerve palsy. Notably, high-grade tumors demonstrated a higher incidence of rapid growth. Diagnostic evaluations encompassed ultrasound (with 50% sensitivity), MRI (with 80% sensitivity), and intraoperative frozen sections (with 88.9% sensitivity). Preoperative suspicion of malignancy was relatively low (31.1%), although there was a higher accuracy for high-grade tumors. TNM staging unveiled T1 as the most prevalent stage (46.7%), while lymph node metastasis occurred in 31.1% of cases, particularly in high-grade and T3 tumors. Significantly, a high tumor stage was associated with a higher likelihood of N+ stage (*s*), and distant metastasis was detected in only one case (M1).

Surgical interventions primarily involved total or subtotal parotidectomy, resulting in complications such as facial nerve palsy (36%), seromas, and salivary fistulas. Neck dissection was undertaken in 67% of cases, revealing occult lymph node metastases in 20%. Resection margins were found to be tumor-free in 56% of cases (R0), and PNI was observed in 37.8%, predominantly in high-grade tumors.

Recurrence was identified in 10 patients (8 locoregional and 2 distant metastases), with a median recurrence-free time of 48.5 months. The 3-year recurrence-free survival (RFS), cancer-specific survival (CSS), and overall survival (OS) rates stood at 72.1%, 91.9%, and 87.5%, respectively. Factors influencing RFS included preoperative facial palsy, T stage, resection margins, and PNI. The tumor stage significantly impacted prognosis, while the N stage did not. Clinical staging (Union for International Cancer Control [UICC] and American Joint Committee of Cancer [AJCC]) revealed a significant difference between Stages I/II and III/IV. Grading did not exhibit a statistically significant effect on RFS.

**Figure 2:**
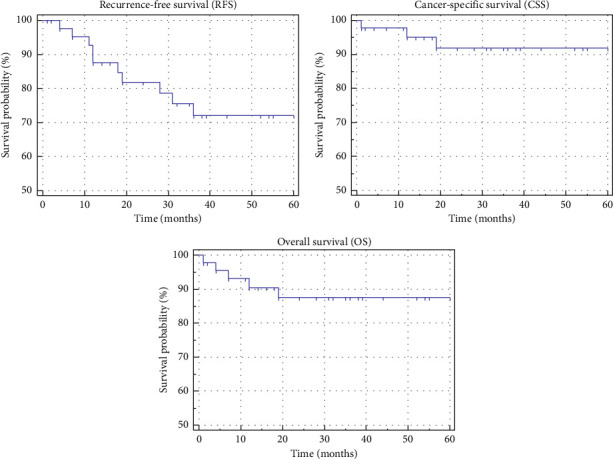
Kaplan–Meier curves of RFS, CSS and OS.

**Figure 3:**
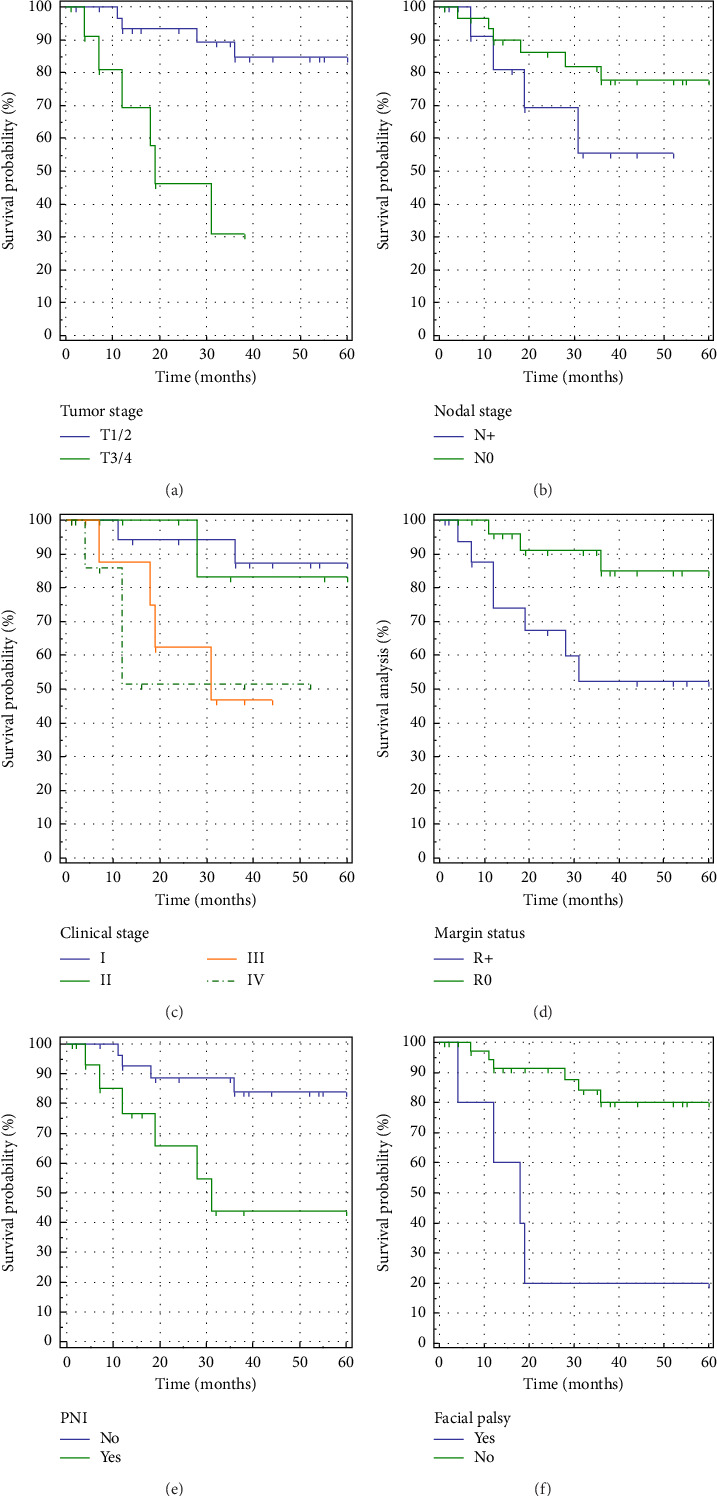
Kaplan–Meier curves on recurrence-free survival according to T stage (a) (*p*=0.0003), N stage (b) (*p*=0.195), clinical stage (c) (*p*=0.039), resection margins (d)(*p*=0.021), perineural invasion (e) (*p*=0.009), and preoperative facial palsy (f) (*p* < 0.0001).

For a detailed overview of patient characteristics, diagnosis, and treatment data, please refer to Tables 1 and 2. In addition, Figures 2 and 3 present survival rates and factors influencing RFS, respectively.

## 4. Discussion

This retrospective single-center study delved into the clinical landscape of 45 patients diagnosed with primary malignant parotid tumors over an 8-year period. The data were meticulously gathered from a nonuniversity tertiary care hospital, specifically the otolaryngology department in Heilbronn, Germany, and its surrounding suburbs, catering to a population of approximately 500,000 in 2023. It is noteworthy that this study holds a unique position as the exclusive analysis of such data within a nonuniversity hospital setting, distinguishing it from more extensive multicenter studies conducted at university hospitals both in Germany and abroad [2, 6–11].

The histological spectrum of the studied cases primarily featured mucoepidermoid carcinomas and squamous cell carcinomas, aligning with the existing research [7, 8, 11–13, 15]. The squamous cell carcinomas showed a higher frequency (22.2%) compared to other studies (0.8%–18%). Primary squamous cell carcinomas of the parotid gland are generally rare and in most of the cases, according to the literature, represent metastases of other tumors of the head and neck. We strictly excluded 28 patients with intraparotideal metastases of tumors of the skin, oral cavity, and lungs and we only included those with no history or clinical signs of other primary tumors.

Consistent with established trends, the mean age of 61 years and a higher incidence in males were in line with previous findings [2, 6, 8, 11–14]. Facial palsy, observed in 11.1%, fell within the range reported in other studies (18%–20%) [6, 8, 16].

Diagnostic methods exhibited varying sensitivity, with intraoperative frozen sections displaying the highest (88.9%), followed by MRI scans (80%), while ultrasounds showed lower sensitivity (50%). These findings align with reports from other investigations [17–19]. FNAB sensitivity was notably low at 11.1%. The potential consideration of core needle biopsy (CNAB) as an alternative to FNAB was suggested based on superior results with no significant complications [20]. However, it is essential to note that this study did not incorporate CNAB in its diagnostic approach.

The staging of tumors unveiled a predominance of lower T1 and T2 stages (Clinical stages I and II), with an incidence of N0 stage at 68.9%, slightly below the reported range of 70%–82% in other studies [6, 7, 11]. Lymph node metastases correlated with higher-grade tumors and advanced T stages, aligning with the more aggressive nature of such tumors. The significance of the T3/T4 stage as a factor for lymph node metastasis was established, and distant metastases, primarily found in the lungs, echoed the patterns observed in the literature [2, 21].

Treatment strategies in this study adhered to the established guidelines, with surgery and adjuvant radiotherapy serving as the primary modalities. Total parotidectomy emerged as the most frequently performed surgery, particularly reserved for T1, T2, or T3 tumors with or without lymph node metastases (Clinical stages I, II, and III). This approach is supported by other studies, which emphasize its effectiveness in addressing intraparotideal lymph node metastases [22–24]. The extent of parotidectomy surfaced as a significant risk factor for postoperative facial palsy [16].

Elective neck dissection was selectively performed even in patients with no apparent risk factors, uncovering occult lymph node metastases, even in low-risk patients with high-grade tumors. This supports the recommendation for elective neck dissection in high-grade tumors, adenocarcinomas, squamous cell carcinomas, and T3/T4 tumors, aligning with the guidelines [3, 5]. The level of neck dissection remains a topic of debate, with varying recommendations present in the literature [25–27].

Resection margins (R0) were consistent with findings in other studies (24%–58%) [6, 7], though higher-stage T3/T4 tumors often resulted in R1 or R2 resections due to their extraparenchymal extension. Postoperative radiation therapy was administered based on recognized risk factors, including high-grade tumors, T3/T4 stage (Clinical stages III and IV), lymph node metastases, positive resection margins, and PNI, aligning with international guidelines [3, 4]. Notably, patients with multiple risk factors who did not receive radiation often did so due to personal refusal or were elderly with comorbidities.

Recurrence rates in this study were comparable to those reported in other studies (11%–41%), with metastases typically detected in the lungs, liver, brain, and bones [1, 2, 6, 7, 10]. The 3-year RFS rate of 72.1% concurs with existing literature (66.7%–83%). Factors influencing RFS included preoperative facial palsy, T stage, resection margins, and PNI, with tumor grade not significantly impacting prognosis within the short-term scope of this study. Extended observation periods may unveil differences, particularly in high-grade tumors.

Patients who underwent less radical treatment exhibited more favorable survival times, primarily attributed to fewer risk factors. However, it is crucial to note that this study did not compare outcomes between chemotherapy and radiation therapy due to the limited number of cases.

OS and tumor-specific survival rates stood at 87.5% and 91.9%, respectively, aligning with findings from previous research (51.4%–88% and 52%–94.2%) [1, 2, 6, 7, 10]. In conclusion, this study conducted in a nonuniversity hospital in Germany has shed light on epidemiological features, diagnostic methods, treatment approaches, and clinical outcomes that are comparable to larger university hospital studies. Limitations include a relatively small sample size, a constrained observation period, and the retrospective nature of the study. Key factors influencing RFS were identified as tumor stage, resection margins, preoperative facial palsy, and PNI. The imperative of early diagnosis, facilitated by improved diagnostic methods, emerged as crucial for achieving tumor-free margins during surgery. Cases presenting with preoperative facial nerve palsy and PNI may warrant a more aggressive therapeutic approach. Prospective studies incorporating specific quality-of-life assessments are essential for a more comprehensive evaluation of the impact of radical therapies.

## 5. Conclusion

In conclusion, this study provides valuable insights into the clinical profile of primary malignant parotid tumors, emphasizing the consistency of our findings with established trends. Our results underscore the significance of accurate diagnostic methods, effective treatment modalities adhering to guidelines, and key prognostic factors influencing RFS. While acknowledging study limitations, our findings contribute to the broader understanding of managing malignant parotid tumors in a nonuniversity hospital setting, warranting further prospective research for optimized therapeutic strategies and improved long-term outcomes.

## Figures and Tables

**Table 1:** Patients' characteristics (*n* = 45).

Characteristic	No. of patients (%)
Gender	
Male	27 (60%)
Female	18 (40%)
Age (years)	
Range	11–91
Average	61
Follow-up (months)	
Range	1–108
Median	43
Histological types	
Mucoepidermoid carcinoma	10 (22.2%)
Squamous cell carcinoma	10 (22.2%)
Salivary duct carcinoma	7 (15.6%)
Acinic cell carcinoma	5 (11.2%)
Adenoid cystic carcinoma	3 (6.8%)
Epithelial–myoepithelial carcinoma	2 (4.4%)
MASC^∗^	2 (4.4%)
Carcinoma ex pleomorphic adenoma	2 (4.4%)
Adenocarcinoma NOS^∗∗^	2 (4.4%)
Lymphoepithelial carcinoma	1 (2.2%)
Myoepithelial carcinoma	1 (2.2%)
T stage	
T1	21 (46.7%)
T2	11 (24.4%)
T3	10 (22.2%)
T4a	2 (4.4%)
T4b	1 (2.2%)
N stage	
N0	31 (68.9%)
N1	7 (15.6%)
N2a	0 (0%)
N2b	6 (13.3%)
N2c	0 (0%)
N3a	0 (0%)
N3b	1 (2.2%)
M stage	
M0	44 (97.8%)
M1	1 (0.2%)
Clinical stage	
I	17 (38%)
II	9 (20%)
III	10 (22%)
IVa	6 (13.3%)
IVb	2 (4.4%)
IVc	1 (2.2%)
Tumor grading	
Low grade	28 (62.2%)
High grade	17 (37.8%)
Perineural invasion (PNI)	
No	28 (62.2%)
Yes	17 (37.8%)
Resection margins	
R0	25 (55.6%)
R+	20 (44.4%)

^∗^MASC: mammary analog secretory carcinoma.

^∗∗^NOS: not otherwise specified.

**Table 2:** Diagnosis, treatment, and outcome data (*n* = 45).

Characteristic	No of patients (%)
Clinical symptoms and signs	
Palpable lump	43 (95.6%)
Rapid growth (< 3 months)	13 (28.9%)
Pain	11 (24.4%)
Palpable lymph nodes	8 (17.8%)
Facial nerve palsy	5 (11.1%)
Fixation to surrounding tissue	5 (11.1%)
Skin infiltration	2 (4.4%)
No symptoms (incidental finding)	1 (2.2%)
Diagnostic methods	
Ultrasound	44 (97.8%)
MRI	15 (33.3%)
CT	3 (6.7%)
Fine-needle aspiration biopsy (FNAB)	9 (20%)
Intraoperative frozen section	9 (20%)
Surgical treatment	
Total/subtotal parotidectomy	23 (51.1%)
Lateral/partial parotidectomy	16 (35.5%)
Radical parotidectomy	6 (13.2%)
Postoperative complications	
Facial nerve palsy	16 (36%)
Seroma	7 (16%)
Salivary fistula	4 (9%)
First bite syndrome	3 (7%)
Frey syndrome	0 (0%)
Other (wound infection and keloid scar)	2 (4%)
Neck dissection	
Yes	30 (67%)
No	15 (33%)
Postoperative radiation therapy	
Radiation therapy	17 (38%)
Chemoradiotherapy	4 (9%)
No radiation therapy	24 (53%)
Recurrence	10 (22.2%)
Locoregional	8 (17.8%)
Distant	2 (4.4%)

## Data Availability

The data supporting the findings of this study are available from the corresponding author upon reasonable request.
